# Identification of Anti-Viral Compounds from *Toxicodendron vernicifluum* Extract That Inhibit the Coronavirus Replication

**DOI:** 10.4014/jmb.2604.04038

**Published:** 2026-06-01

**Authors:** Jihun Choi, Jang Hoon Kim, Siyun Lee, Chunghyeon Lee, Seungju Cho, Sumin Kim, Eunjin Cho, Jayhyun Park, Yu Jin Kim, Ik Soo Lee, Junsoo Park

**Affiliations:** 1 Division of Biological Science and Technology, Yonsei University, Wonju 26493, Republic of Korea; 2 Department of Herbal Crop Research, National Institute of Horticultural & Herbal Science, RDA, Eumsung 27709, Republic of Korea; 3 Analysis & Evaluation department, Korea Mine Rehabilitation and Mineral Resources Corporation, Wonju 26464, Republic of Korea; 4 Department of Analysis and Assessment, Korea Mine Rehabilitation and Mineral Resources Corporation, Wonju 26464, Republic of Korea; 5 Km Convergence Research Division, Korea Institute of Oriental Medicine, Daejeon, 34054, Republic of Korea

**Keywords:** Coronavirus, *Toxicodendron vernicifluum*, Antiviral, Fisetin, HCoV-OC43

## Abstract

Coronavirus can cause diseases ranging from mild cough to severe COVID-19. Although vaccines and antiviral agents for coronaviruses are available, alternative strategies are still needed to address emerging variants in the future. *Toxicodendron vernicifluum* is known to be rich in flavonoids, which exhibit antioxidant, anti-inflammatory, anticancer, and antiviral activities. However, the antiviral activity of *T. vernicifluum* against coronaviruses has not been fully investigated. Here, we report that the *T. vernicifluum* ethanol extract (TVE) shows antiviral effects against human coronavirus. TVE treatment inhibited coronavirus replication as well as viral infectivity. To elucidate the active constituents responsible for this activity, comprehensive phytochemical profiling of TVE was performed using UPLC-Q-Orbitrap-MS, leading to the tentative identification of major phytochemicals. Based on this analytical profiling, individual compounds were isolated from TVE and evaluated for antiviral activity against coronavirus. We found that ethyl gallate, fisetin, butin, sulfuretin, and gallic acid exhibit antiviral activity against coronavirus.

## Introduction

Coronaviruses represent a persistent and evolving threat to human health, causing diseases that range from self-limiting upper respiratory infections to severe and life-threatening illnesses such as COVID-19 [1, 2]. Human coronaviruses are estimated to account for approximately 15–30% of common cold cases worldwide, with four endemic strains—HCoV-229E, HCoV-NL63, HCoV-OC43, and HCoV-HKU1—circulating continuously in the global population [3, 4]. Notably, HCoV-OC43 belongs to the Betacoronavirus genus, together with SARS-CoV-2, and has been widely used as an experimental model to investigate coronavirus biology and antiviral strategies [5-7].

Unlike DNA viruses, coronaviruses possess RNA genomes with high mutation rates, enabling rapid viral evolution and facilitating the emergence of immune-evasive variants [8, 9]. Despite the development of vaccines against SARS-CoV-2, the continual appearance of novel variants underscores the limitations of vaccine-based control alone and raises concerns about recurrent infections resembling seasonal coronavirus outbreaks [10-12]. These challenges highlight the need for alternative antiviral therapeutics to achieve sustained control of coronavirus-associated diseases [13, 14].

*Toxicodendron vernicifluum* (syn. *Rhus verniciflua*, known in Korea as “Ot”) is a traditional medicinal plant that exhibits broad pharmacological activities, including anti-inflammatory, antioxidant, anticancer, and antiviral effects [15-17]. *T. vernicifluum* extract contains the allergen urushiol, which can induce allergic reactions; therefore, allergen-removed (urushiol-free) extracts are often used to examine its pharmacological effects [18, 19]. Several reports support that *T. vernicifluum* has antiviral activity against various viruses. A previous study showed that the ethyl acetate fraction of *T. vernicifluum* inhibited the neuraminidase activity of influenza A virus and fish pathogenic viruses [20, 21]. In addition, urushiol derivatives also showed inhibitory activity against the reverse transcriptase of human immunodeficiency virus (HIV) [22].

In this study, we aimed to identify plant extracts with antiviral activity and found that *T. vernicifluum* ethanol extract effectively inhibits the replication of human coronavirus. To investigate the phytochemical basis of this antiviral efficacy, we characterized the chemical profile of TVE using UPLC-Q-Orbitrap-MS, which identified 14 major constituents, including various flavonoids and phenolic compounds. Guided by these high-resolution mass spectrometry analyses, we isolated individual compounds and demonstrated that several of the isolated compounds exhibit antiviral activity against human coronavirus.

## Materials and Methods

### Preparation of *T. vernicifluum* Extract (TVE)

In August 2023, *T. vernicifluum* heartwood was obtained from a domestic herbal vendor in the Republic of Korea and botanically authenticated by Dr. J.H. Kim. A voucher specimen (TV 23) was deposited in the herbarium of the National Institute of Horticultural and Herbal Science (Department of Herbal Crop Research, Republic of Korea). To prepare the extract, the air-dried plant material (5.0 kg) was macerated in 95% aqueous ethanol (2 × 36 L) for 48 h at 60°C. The resulting ethanolic suspension was filtered through filter paper and subsequently concentrated under reduced pressure to yield 208 g of the final extract, designated as TVE.

### UPLC-Q-Orbitrap-MS Analysis

Metabolite profiling of TVE was performed using a Thermo Fisher Scientific Dionex UltiMate 3000 system coupled to a Q-Exactive Orbitrap mass spectrometer. Analytes were isolated on an Acquity BEH C18 column (100 × 2.1 mm, 1.7 μm; Waters) through gradient elution using 0.1% formic acid in both water and acetonitrile. Detection was conducted in negative ion mode. The HESI source conditions were set as follows: capillary temperature, 350°C; spray voltage, 3.5 kV; and S-lens RF level, 50. The sheath and auxiliary gas flow rates were maintained at 40 and 10 arbitrary units, respectively. Data were acquired using a full MS/dd-MS² method. The full scan MS parameters included a resolution of 70,000, a scan range of m/z 100–1500, and a maximum injection time of 100 ms in profile mode. Data-dependent MS² parameters were set to a resolution of 17,500, normalized collision energy (NCE) of 25 eV, maximum injection time of 50 ms, loop count of 10, and MSX count of 1. All data acquisition and processing were performed using Xcalibur and TraceFinder 5.1 software.

### Coronavirus Infection

HCoV-OC43 was acquired from ATCC (USA), while rhabdomyosarcoma (RD) cells were sourced from the Korean Cell Line Bank (Republic of Korea). RD cells were cultured in DMEM (Republic of Korea) with the addition of 10% FBS (Thermo Fisher Scientific, USA) and 1% penicillin–streptomycin (Welgene). RD cells were infected with human coronavirus (10^6^ PFU/mL) as previously described [5]. Briefly, cells were incubated with the indicated dilutions of medium containing coronavirus at a multiplicity of infection (MOI) of 0.01, and the infected cells were maintained in MEM supplemented with 2% fetal bovine serum (FBS).

To promote plaque formation, the conditioned medium was harvested 72 h post-infection and then passed through 0.45 μm CA membrane filters (Sartorius, Germany) for filtration. RD cells were cultured in 12-well plates and subsequently treated with varying concentrations of conditioned medium containing coronavirus. Infected cells were incubated for an additional 4 days at 33°C to facilitate plaque formation, then fixed with 4% paraformaldehyde and stained using a 0.2% crystal violet solution. The assessment of cell viability was conducted using an MTT assay, as outlined in prior methodologies [23].

### Western Blot

The expression levels of coronavirus proteins were assessed through Western blot analysis utilizing an anti-HCoV-OC43 antibody. The cells and conditioned medium were separately collected and then lysed using a cell lysis buffer composed of 50 mM NaCl, 50 mM HEPES at pH 7.5, and 1% NP-40, with the addition of a protease inhibitor cocktail. Proteins in equal amounts were separated using SDS-PAGE and then transferred to PVDF membranes (Cytiva, USA). The membranes were blocked using 3% skim milk in TBS containing 0.1% Tween-20 (TBS-T) and subsequently probed with an anti-HCoV-OC43 antibody (Sigma-Aldrich, USA). Immunoreactive bands were detected using a ChemiDoc Imaging System (Bio-Rad). Mouse polyclonal anti-HCoV-OC43 antibodies were generated using inactivated HCoV-OC43 particles in mice [24].

### Quantitative RT-PCR

Quantitative RT-PCR was conducted to assess coronavirus RNA levels in both cells and conditioned medium. Cells and medium were collected separately, and total RNA was extracted using the PURE™ Total RNA Extraction Kit (Infusion Tech, Republic of Korea) following the manufacturer's guidelines. An equal quantity of total RNA was employed for the synthesis of complementary DNA (cDNA) using the M-MLV cDNA Synthesis Kit (Enzynomics, Republic of Korea). qRT-PCR was performed using a QuantStudio 3 Real-Time PCR System (Thermo Fisher Scientific), with RPL4 used as an internal control. Primer sequences for viral and host genes were described previously [5].

### Scanning Electron Microscopy (SEM)

For scanning electron microscopy (SEM), RD cells were cultured on sterilized 9-mm coverslips and infected with HCoV-OC43 for 3 days. Cells were fixed using 2.5% glutaraldehyde for a duration of 1 hour and subsequently dehydrated through a graded ethanol series comprising concentrations of 20%, 40%, 60%, 80%, 90%, and 100%. Following dehydration, the samples were subjected to air-drying within a vacuum desiccator for a duration of 1 h. The desiccated cells were coated with platinum, and scanning electron microscopy (SEM) images were obtained using a SUPRA 40 scanning electron microscope (Carl Zeiss, Germany).

### Statistical Analysis

The data obtained from Western blot, quantitative RT-PCR, and MTT assays were subjected to analysis using two-tailed Student’s t-tests in Excel (Microsoft, USA). IC_50_ values were calculated using the AAT Bioquest IC_50_ Calculator (AAT Bioquest, USA), and bar graphs were generated using GraphPad Prism 8 (GraphPad Software, USA). Statistical significance was determined using a *p*-value of less than 0.05.

## Results

### *T. vernicifluum* Ethanol Extract (TVE) Treatment Reduces the Expression of Human Coronavirus in the Conditioned Medium

**Fig. 1:**
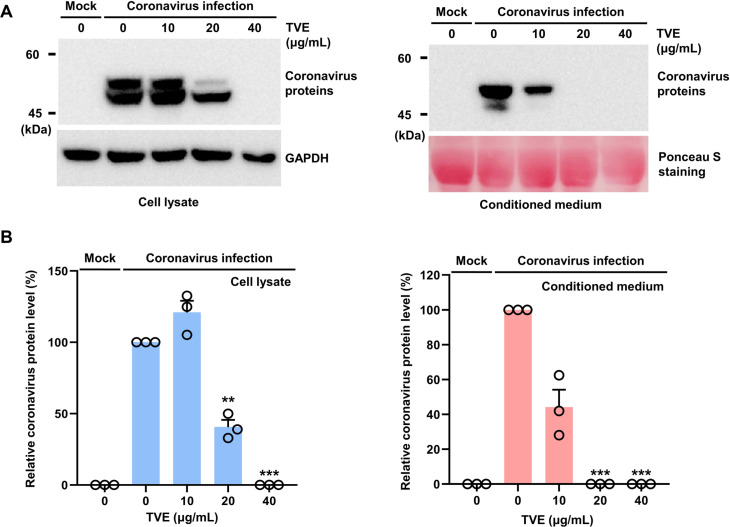
*T. vernicifluum* ethanol extract (TVE) inhibits the expression of coronavirus proteins. (**A**) RD cells were infected with human coronavirus OC43 (HCoV-OC43) and subsequently treated with TVE. Western blot analysis was performed on cell lysates and conditioned medium to assess the expression of coronavirus proteins. GAPDH and Ponceau S staining were used as loading controls for the cell lysates and conditioned media, respectively. (**B**) Coronavirus protein expression levels were quantified and presented in the graph. Significance levels for the comparison between the infected group and TVE treatment are indicated as follows: *p < 0.05, **p < 0.01, ***p < 0.001.

To identify antiviral compounds from plants, we screened various plant extracts for antiviral activity. During this screening, we found that *T. vernicifluum* ethanol extract (TVE) exhibited antiviral activity against human coronavirus. When RD cells were infected with human coronavirus OC43 (HCoV-OC43), a marked increase in coronavirus protein expression was observed (Fig. 1A). Treatment with TVE significantly reduced the expression level of coronavirus proteins in infected cells (Fig. 1B).

We also examined coronavirus protein expression in the conditioned medium, which contains released virion particles. TVE treatment reduced coronavirus protein levels in the conditioned medium, and viral proteins were barely detectable (Fig. 1B). Quantification of coronavirus protein expression in the conditioned medium revealed that TVE treatment decreased viral protein levels in a dose-dependent manner (Fig. 1). These results indicate that TVE inhibits coronavirus replication and suggest that TVE contains antiviral phytochemicals.

### TVE Treatment Inhibits the Replication of Human Coronavirus

**Fig. 2:**
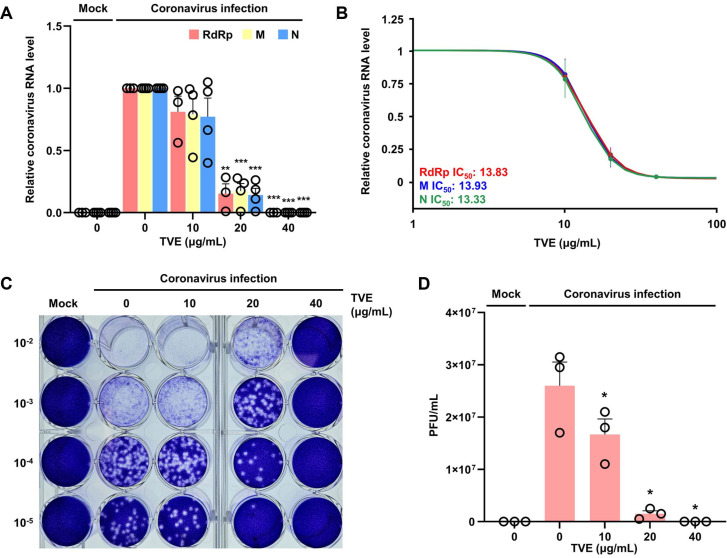
TVE treatment reduces the release of coronavirus. (**A**) RD cells were infected with coronavirus, and the relative amounts of coronavirus RNA in the conditioned medium were analyzed by qRT-PCR. (**B**) IC_50_ values were calculated based on qRT-PCR results. (**C**) TVE treatment reduces infectious coronavirus particles. RD cells were infected with coronavirus, followed by vehicle or TVE treatment. The conditioned medium was used for plaque formation assays. (**D**) Plaque numbers were counted and presented in the graph. Significance levels for the comparison between the infected group and TVE treatment are indicated as follows: *p < 0.05, **p < 0.01, ***p < 0.001.

Coronaviruses in the conditioned medium are produced and released from infected cells; therefore, we examined whether TVE treatment decreases coronavirus replication. Cells were infected, followed by TVE treatment, and the conditioned medium was collected to measure the levels of coronavirus RNA. We analyzed the RNA levels of membrane protein (M), nucleoprotein (N), and RNA-dependent RNA polymerase (RdRp). Quantitative RT-PCR showed that TVE treatment decreased coronavirus RNA levels in a dose-dependent manner (Fig. 2A). We also determined the half-maximal inhibitory concentration (IC_50_), with the IC_50_ values for M, N, and RdRp ranging from 13.33 to 13.93 μg/mL (Fig. 2B).

Next, we examined the infectivity of coronavirus following TVE treatment using a plaque formation assay. While vehicle treatment resulted in a large number of plaques, TVE treatment decreased plaque numbers in a dose-dependent manner (Figs. 2C and 2D). These results indicate that TVE treatment reduces the number of infectious particles and inhibits coronavirus replication.

### TVE Treatment Reduces Coronavirus Particles

**Fig. 3:**
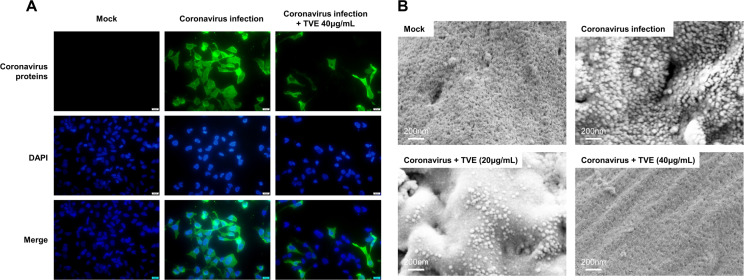
TVE treatment decreases the number of infected cells and coronavirus particles on the infected cell membrane. (**A**) TVE treatment decreases the number of infected cells. RD cells were infected with coronavirus and subsequently treated with either a vehicle or TVE. Immunofluorescence images show coronavirus protein expression (green) with nuclear counterstaining by DAPI (blue). The bottom row displays merged images. Scale bars, 20 μm. (**B**) TVE treatment decreases coronavirus particles in infected cells. Scanning electron microscopy images show the morphology of infected cells. Mock-treated cells exhibit smooth surfaces, whereas coronavirus-infected cells display viral particles on the cell surface. TVE treatment reduces surface-associated viral particles. Scale bars, 200 nm.

We next examined whether TVE treatment reduced the number of infected cells. RD cells were infected with coronavirus and subsequently treated with either a vehicle or TVE. While vehicle-treated cells showed extensive expression of coronavirus proteins, TVE treatment markedly reduced coronavirus protein expression (Fig. 3A). These results indicate that TVE treatment reduces coronavirus infection.

Because the plaque formation assay demonstrated that coronavirus infectivity was decreased by TVE treatment, we sought to directly observe the produced coronavirus particles in infected cells. RD cells were infected with human coronavirus and treated with TVE, followed by fixation and observation using scanning electron microscopy (SEM). Coronavirus infection resulted in the production of viral particles on the membranes of infected cells, whereas TVE treatment reduced the number of coronavirus particles on the cell membrane (Fig. 3B). These results indicate that TVE treatment decreases the production of coronavirus particles in infected cells.

### Identification of Antiviral Single Compounds from TVE

**Fig. 4:**
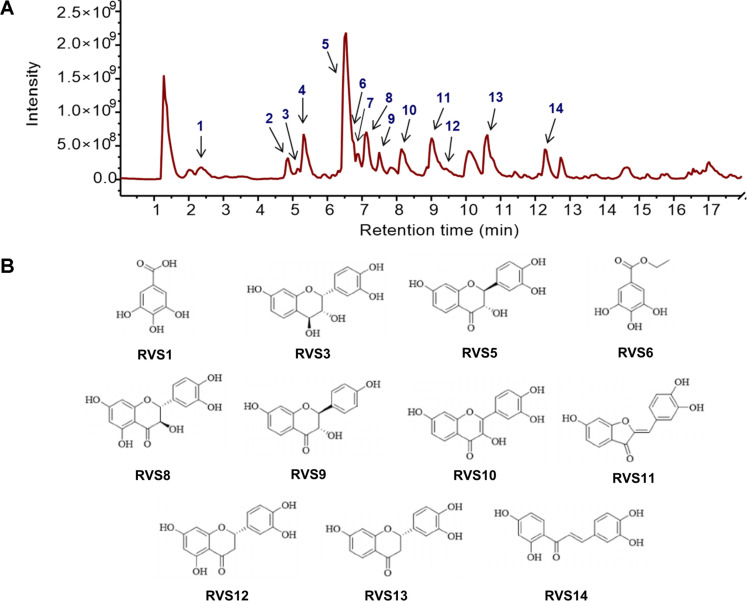
UPLC-Q-Orbitrap-MS chromatogram of TVE. (**A**) The total ion chromatogram (TIC) of TVE was acquired in negative electrospray ionization mode. The numbered peaks (1–14) correspond to the tentatively identified RVS compounds listed in Table 1. (**B**) The structures of selected chemicals used for further study are shown. RVS numbers correspond to the peak order based on retention time.

To identify the active constituents responsible for the antiviral properties of TVE, its chemical profile was characterized using UPLC-Q-Orbitrap-MS in negative ion mode (Fig. 4A). A total of 14 major compounds were tentatively identified by comparing their retention times, accurate mass data (mass error < 5 ppm), and MS/MS fragmentation patterns with reference standards and spectral databases (Table 1). Each peak was numbered as RVS1–14, where RVS stands for *Rhus verniciflua* Stokes, another name for *Toxicodendron vernicifluum*. The RVS numbers correspond to the order of the peaks according to their retention times. Based on their chemical scaffolds, the identified compounds were primarily classified into two major categories: phenolic acids and flavonoids. The phenolic group included gallic acid (RVS1) and its derivatives, such as methyl gallate (RVS4), ethyl gallate (RVS6), and the gallotannin 1,2,3,4,6-pentagalloylglucose (RVS7). The flavonoid group comprised the majority of identified constituents, including flavanones (eriodictyol-7-O-glucoside, eriodictyol, and butin; RVS2, 12, 13), flavanonols (fustin, taxifolin, and garbanzol; RVS5, 8, 9), flavonols (fisetin; RVS10), aurones (sulfuretin; RVS11), chalcones (butein; RVS14), and flavan-3,4-diols (fisetinidol-4-ol; RVS3) (Fig. 4B). Although the quantification of specific flavonoids and phenolics in TVE was reported in our previous study [25], verifying their presence in the current extract was necessary to pinpoint the active antiviral agents. Accordingly, based on this profiling, a subset of previously isolated RVS compounds (RVS1, RVS3, RVS5, RVS6, and RVS8–14) was selected to evaluate their individual inhibitory effects on coronavirus replication.

**Fig. 5:**
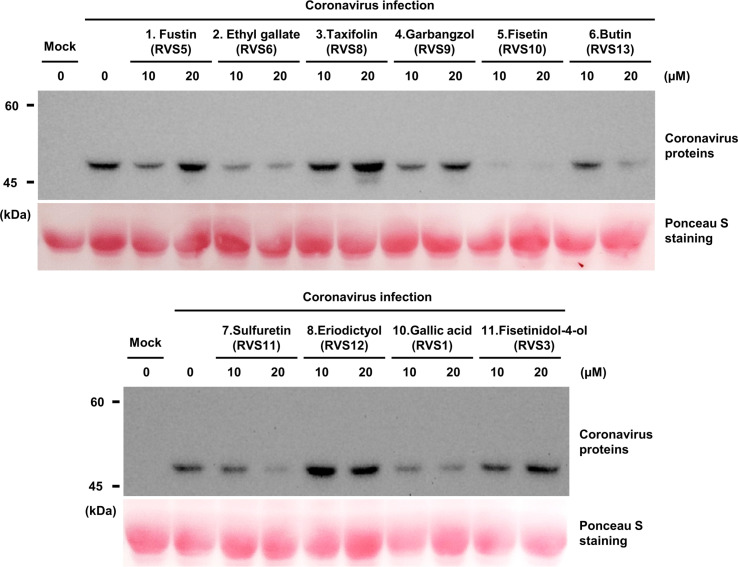
Screening of antiviral compounds from TVE. RD cells were infected with coronavirus and subsequently treated with isolated single compounds derived from TVE. The conditioned medium was subjected to Western blot analysis to examine antiviral effects. Ethyl gallate, fisetin, butin, sulfuretin, and gallic acid showed antiviral activity against coronavirus.

Two concentrations (10 μM and 20 μM) were applied to coronavirus-infected cells, and the expression levels of coronavirus proteins were examined (Fig. 5). During the experiments, butein (RVS14) was excluded due to strong cytotoxicity in infected cells. Several compounds were found to effectively decrease the expression of coronavirus proteins, including gallic acid (RVS1), ethyl gallate (RVS6), fisetin (RVS10), sulfuretin (RVS11), and butin (RVS13). Interestingly, the expression levels of coronavirus proteins in cell lysates were not significantly reduced by treatment with individual compounds; however, coronavirus protein levels in the conditioned medium were markedly decreased following treatment (Fig. 5). Because coronavirus proteins detected in the conditioned medium originate from virion particles produced and released from infected cells, these results suggest that these individual compounds inhibit coronavirus production in cells.

### Fisetin Inhibits the Replication of Human Coronavirus

**Fig. 6:**
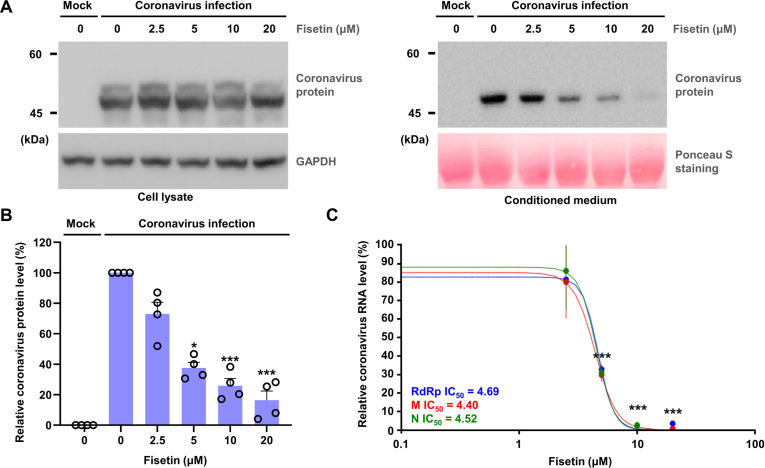
Fisetin shows antiviral effects against coronavirus. (**A**) Fisetin treatment decreased coronavirus protein expression. Coronavirus-infected cells were treated with the indicated concentrations of fisetin. Western blot analysis was performed on cell lysates and conditioned medium, depicted in the left and right panels, respectively. (**B**) Coronavirus protein levels in the conditioned medium were quantified and presented in the graph. (**C**) Fisetin treatment decreased coronavirus RNA levels. Coronavirus RNA in the conditioned medium was quantified and presented in the graph, and IC_50_ values were calculated. Significance levels for the comparison between the infected group and TVE treatment are indicated as follows: *p < 0.05, **p < 0.01, ***p < 0.001.

Among the isolated single compounds, fisetin showed the strongest antiviral effect. We further confirmed the antiviral activity of fisetin against human coronavirus. Although fisetin has been reported to inhibit SARS-CoV-2, its antiviral activity against the HCoV-OC43 strain has not been examined [26, 27]. Treatment of coronavirus-infected cells with fisetin did not significantly reduce coronavirus protein levels in cell lysates; however, fisetin markedly inhibited viral protein expression in the conditioned medium (Figs. 6A and 6B). In addition, fisetin treatment reduced coronavirus RNA levels in the conditioned medium (Fig. 6C). The calculated IC_50_ of fisetin ranged from 4.40 to 4.69 μM, which was substantially lower than that of TVE, indicating that fisetin exhibits stronger inhibitory activity than the crude extract.

## Discussion

*Toxicodendron vernicifluum* (known in Korea as “Ot”) is a traditional medicinal plant that exhibits broad pharmacological activities [15]. *T. vernicifluum* is also commonly used as a food ingredient in Korea, where it is often cooked with chicken or duck. Although antiviral effects of *T. vernicifluum* against influenza virus have been reported, its antiviral activity against coronavirus has not been studied [20]. To our knowledge, this represents the first documented evidence that *T. vernicifluum* possesses antiviral properties against human coronavirus.

In this study, we isolated antiviral compounds that exhibit inhibitory effects against human coronavirus. Screening results demonstrated that ethyl gallate, fisetin, butin, sulfuretin, and gallic acid inhibited the expression of coronavirus proteins (Fig. 5). Among these, fisetin treatment resulted in the most significant reduction of coronavirus proteins, and we further demonstrated that fisetin inhibits both coronavirus protein expression and viral RNA levels (Fig. 6). Previous reports have shown that fisetin inhibits SARS-CoV-2 replication and suppresses 3CL protease activity [26, 27]. Ethyl gallate, gallic acid, sulfuretin, and butin also exhibited relatively weaker inhibitory activity against human coronavirus (Fig. 5). According to previous reports, ethyl gallate and gallic acid have been shown to inhibit coronavirus replication [28, 29]. In addition, in silico analyses predicted that butin and sulfuretin are potential inhibitors of coronavirus enzymes [30, 31].

Taken together, these results support that *T. vernicifluum* ethanol extract exerts antiviral effects against coronavirus through these bioactive components. Moreover, we speculate that fisetin is a major active compound responsible for the antiviral activity of TVE against human coronavirus. Fisetin treatment dramatically changed the level of coronavirus protein in the conditioned media; however, the level of viral protein in the cell lysates was not significantly changed (Fig. 6). We speculate that fisetin may interfere with later stages of viral replication, such as viral release, which could explain why intracellular coronavirus protein expression was not markedly altered. Further studies will be required to clarify the difference in coronavirus protein expression between the conditioned media and cell lysates.

*T. vernicifluum* is a medicinal plant traditionally used in China, Japan, and Korea. However, urushiol, a component of *T. vernicifluum*, is allergenic, and urushiol-free extracts are often used to examine its pharmacological effects. Urushiol derivatives have been reported to exhibit antiviral activity against human immunodeficiency virus [22], whereas our results demonstrate that components other than urushiol show antiviral effects against human coronavirus. Therefore, urushiol-free (allergen-free) extracts of *T. vernicifluum* may be used as potential antiviral agents against coronavirus.

In addition, chicken soup prepared with *T. vernicifluum* (“ot-dak” in Korean) is popular in Korea and is traditionally believed to improve physical strength and alleviate symptoms of the common cold. Because a significant proportion of common cold cases are caused by coronavirus infections [3], it is possible that *T. vernicifluum* used as a food ingredient may help alleviate common cold symptoms by inhibiting coronavirus replication. Further studies evaluating *T. vernicifluum* as a functional food additive are required to establish a direct relationship with its antiviral effects.

## Supplemental Materials

Supplementary data for this paper are available on-line only at http://jmb.or.kr.



## Figures and Tables

**Table 1:** Identification of major phytochemical constituents in the TVE using UPLC-Q-Orbitrap-MS in negative ion mode.

ID	RT (min)	Calculated (m/z)	Estimated (m/z)	Error (ppm)	Adduct	Chemical formula	MS/MS Fragments (m/z)	Identification
RVS1	2.38	169.0142	169.0137	-2.958	[M-H]^-^	C_7_H_6_O_5_	169.01, 125.02, 81.03	Gallic acid (**10**)
RVS2	4.86	449.1090	449.1086	-0.820	[M-H]^-^	C_21_H_22_O_11_	287.06, 151.00	Eriodictyol-7-O-glucoside
RVS3	5.13	289.0718	289.0716	-0.692	[M-H]^-^	C_15_H_14_O_6_	271.06, 151.00, 137.02	Fisetinidol-4-ol (**11**)
RVS4	5.35	183.0291	183.0289	-1.175	[M-H]^-^	C_8_H_8_O_5_	183.03, 124.01, 82.00	Methyl gallate
RVS5	6.54	287.0561	287.0559	-0.738	[M-H]^-^	C_15_H_12_O_6_	269.04, 163.00, 91.02	Fustin (**1**)
RVS6	6.72	197.0456	197.0452	-2.030	[M-H]^-^	C_9_H_10_O_5_	197.02, 169.04	Ethyl gallate (**2**)
RVS7	6.85	939.1109	939.1106	-0.319	[M-H]^-^	C_41_H_32_O_26_	769.12, 169.03	1,2,3,4,6-Pentagalloylglucose
RVS8	7.12	303.0510	303.0509	-0.330	[M-H]^-^	C_15_H_12_O_7_	285.03, 125.04	Taxifolin (**3**)
RVS9	7.42	271.0612	271.0611	-0.332	[M-H]^-^	C_15_H_12_O_5_	271.06, 253.05, 119.05	Garbanzol (**4**)
RVS10	8.13	285.0405	285.0404	-0.351	[M-H]^-^	C_15_H_10_O_6_	285.02, 163.03, 135.02, 121.04	Fisetin (**5**)
RVS11	9.05	269.0455	269.0455	-0.112	[M-H]^-^	C_15_H_10_O_5_	269.04, 241.05, 133.03	Sulfuretin (**7**)
RVS12	9.46	287.0561	287.0558	-1.045	[M-H]^-^	C_15_H_12_O_6_	287.12, 151.03, 135.04	Eriodictyol (**8**)
RVS13	10.59	271.0601	271.0598	-1.107	[M-H]^-^	C_15_H_10_O_5_	271.06, 135.04	Butin (**6**)
RVS14	12.34	271.0612	271.0608	-1.476	[M-H]^-^	C_15_H_12_O_5_	271.06, 135.04, 119.05, 109.02	Butein (**9**)
