# Supplementary figures and images for: Identification of Anti-Viral Compounds from Toxicodendron vernicifluum Extract That Inhibit the Coronavirus Replication
Source: J Microbiol Biotechnol. 2026 Jun 1;36:e2604038. doi: 10.4014/jmb.2604.04038 (PMC13253540; doi:10.4014/jmb.2604.04038)

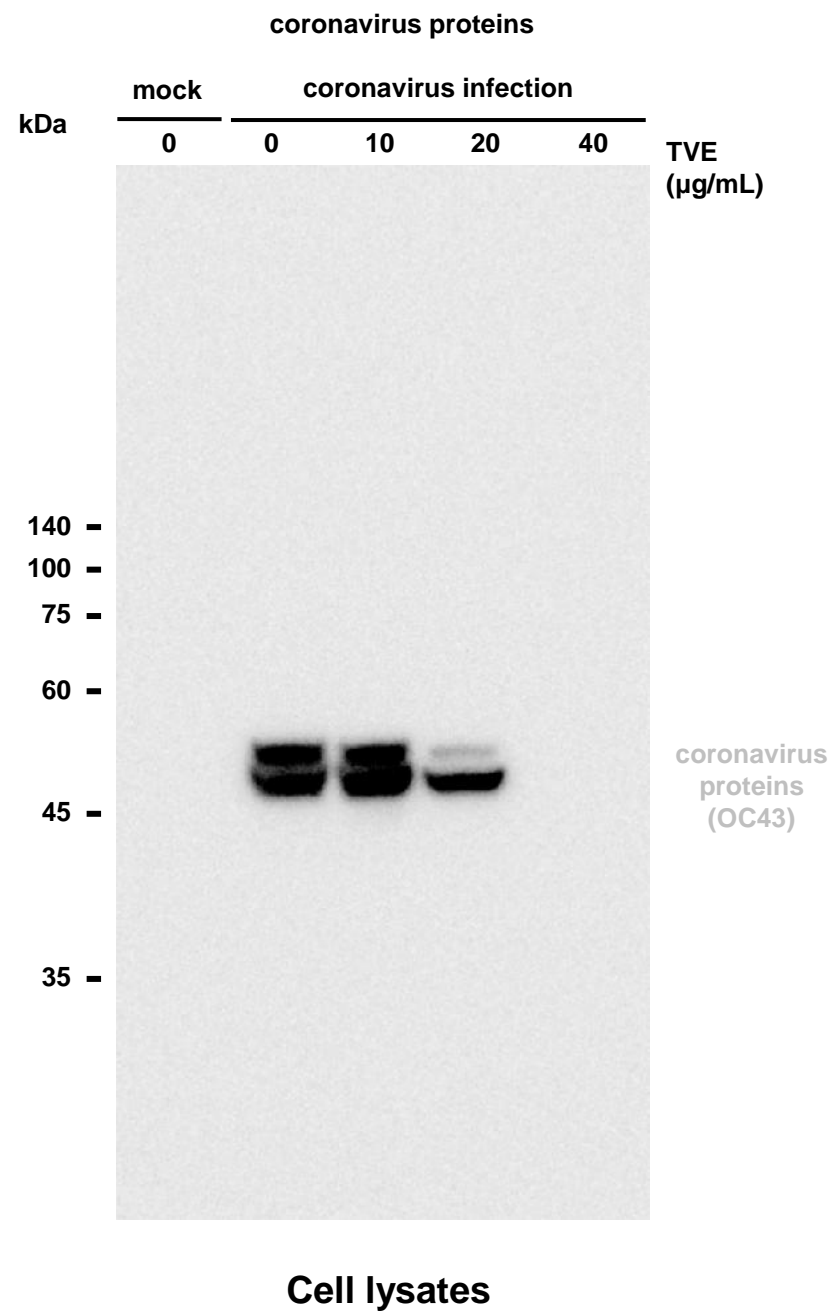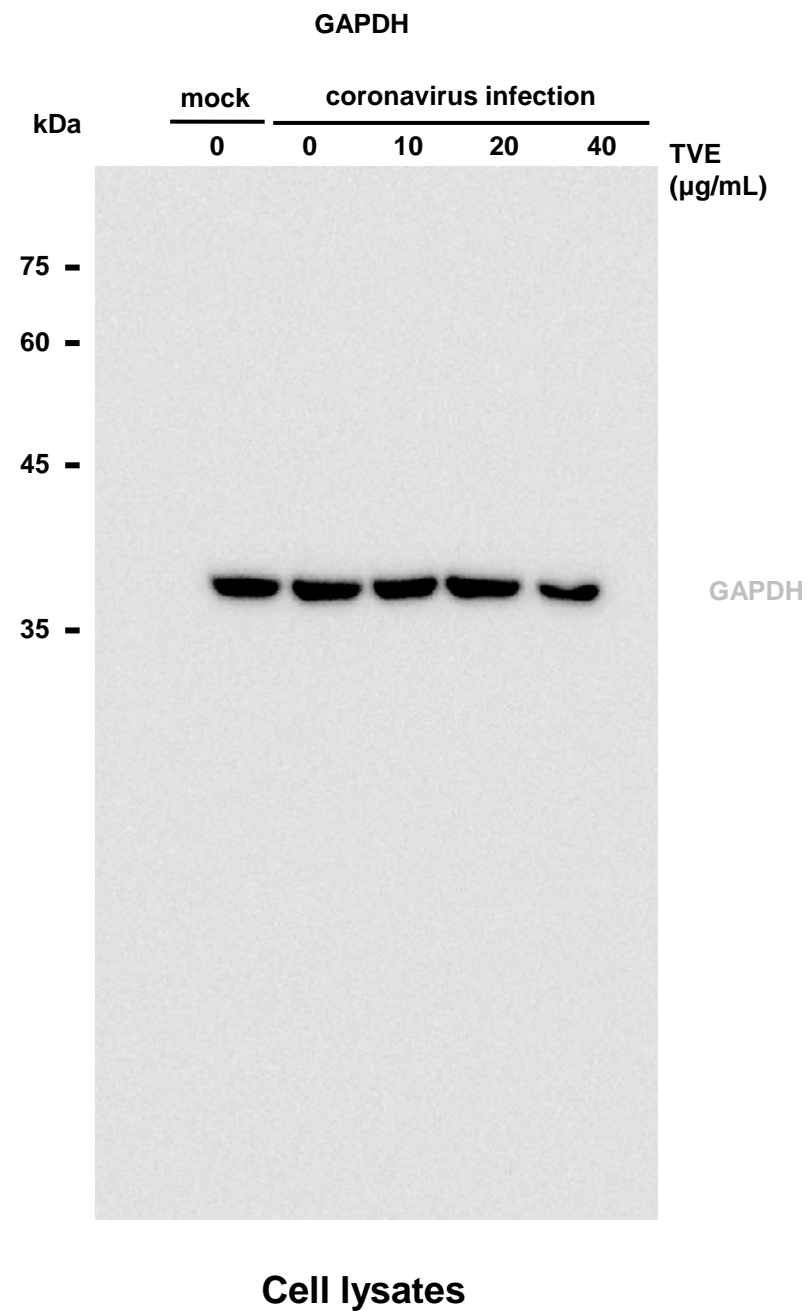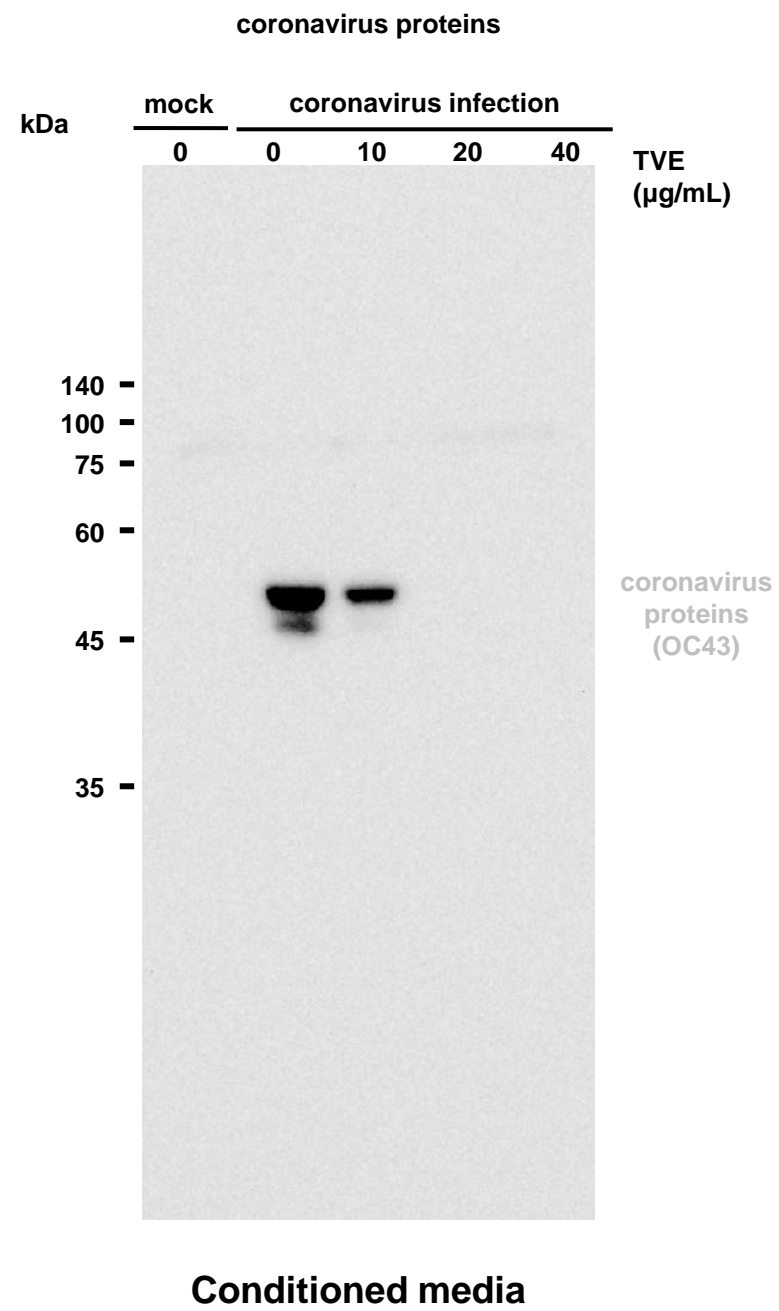

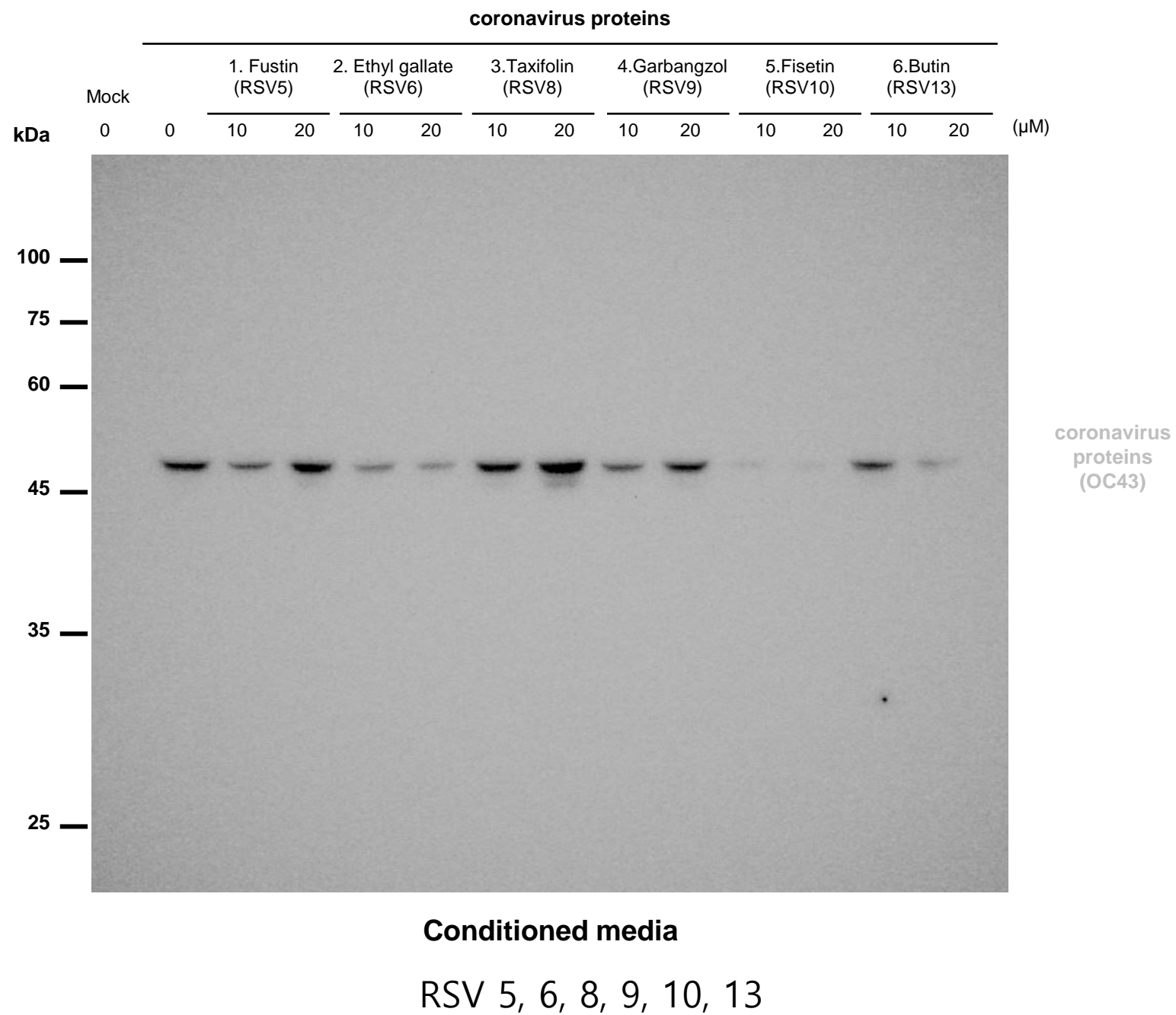

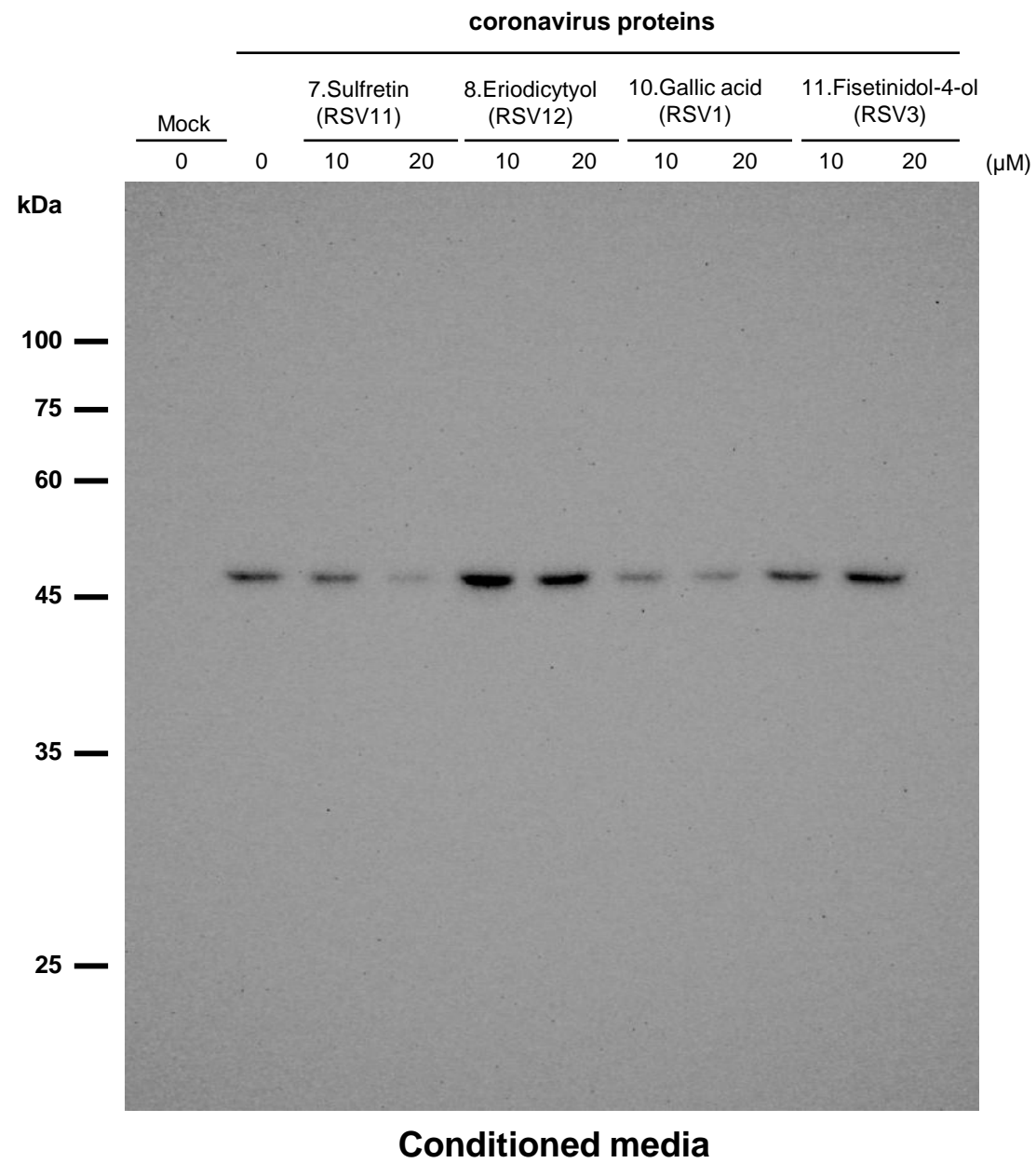

RSV11,13,1,3

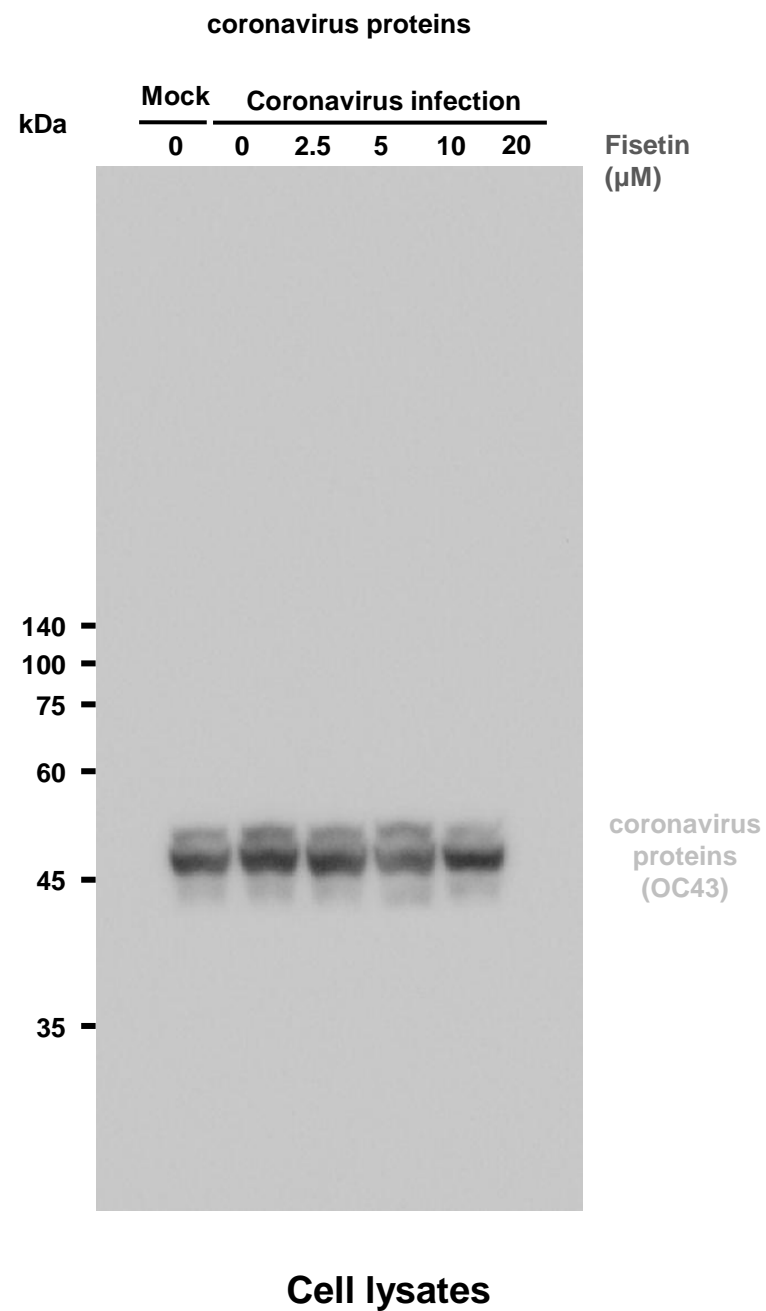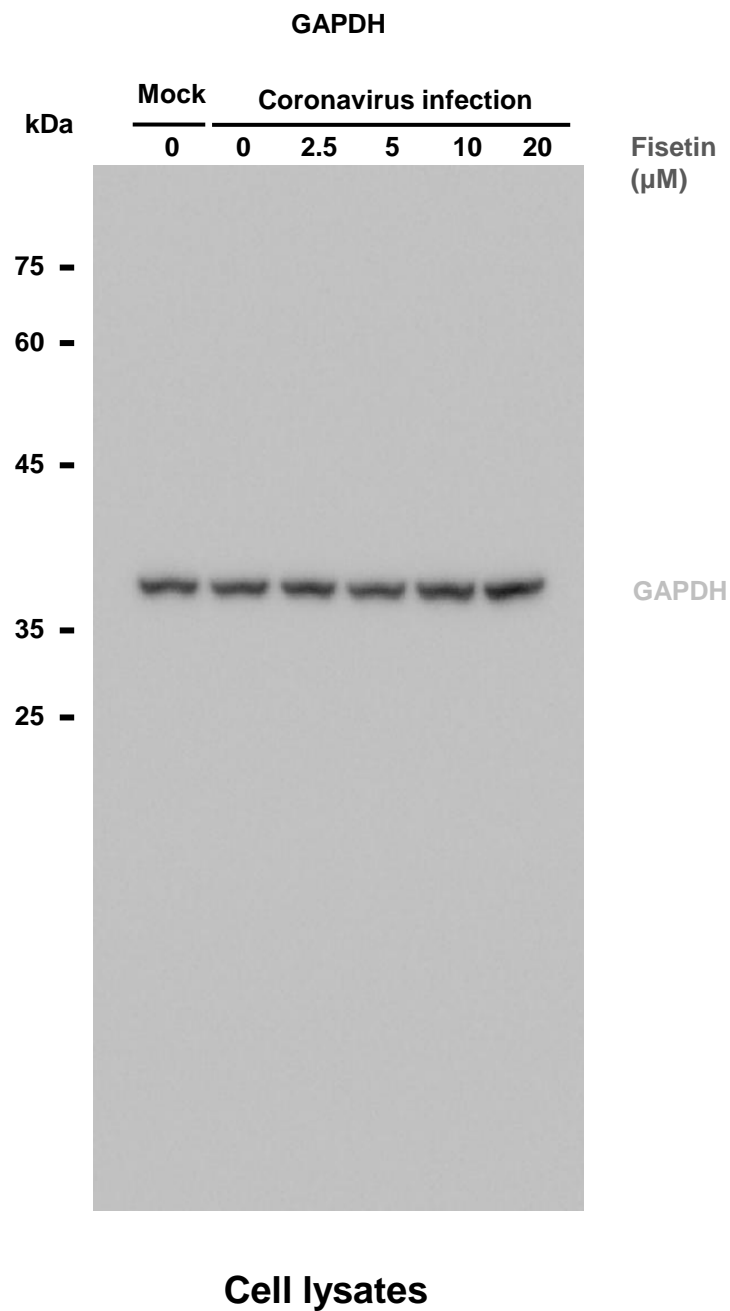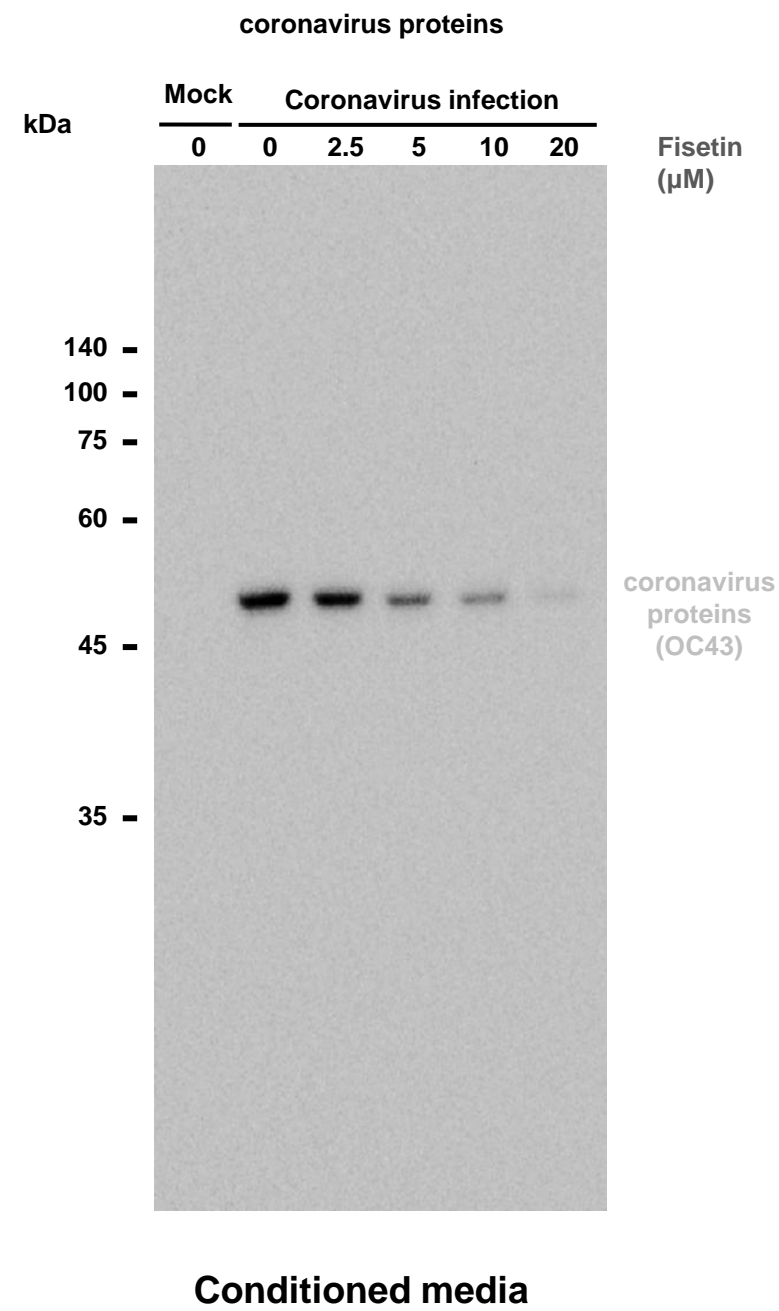

Supplement: Supplementary file 1 [file jmb-36-e2604038-supple.pdf]
